# BrainCDNet: a concatenated deep neural network for the detection of brain tumors from MRI images

**DOI:** 10.3389/fnhum.2024.1405586

**Published:** 2024-06-11

**Authors:** K. Rasool Reddy, Kandala N. V. P. S. Rajesh, Ravindra Dhuli, Vuddagiri Ravi Kumar

**Affiliations:** ^1^Department of ECE, NRI Institute of Technology (Autonomous), Vijayawada, India; ^2^School of Electronics Engineering, VIT-AP University, Andhra Pradesh, India; ^3^Department of ECE, Koneru Lakshmaiah Education Foundation, Hyderabad, India

**Keywords:** brain tumors, deep learning, magnetic resonance imaging, Nimble filter, normalization

## Abstract

**Introduction:**

Brain cancer is a frequently occurring disease around the globe and mostly developed due to the presence of tumors in/around the brain. Generally, the prevalence and incidence of brain cancer are much lower than that of other cancer types (breast, skin, lung, etc.). However, brain cancers are associated with high mortality rates, especially in adults, due to the false identification of tumor types, and delay in the diagnosis. Therefore, the minimization of false detection of brain tumor types and early diagnosis plays a crucial role in the improvement of patient survival rate. To achieve this, many researchers have recently developed deep learning (DL)-based approaches since they showed a remarkable performance, particularly in the classification task.

**Methods:**

This article proposes a novel DL architecture named BrainCDNet. This model was made by concatenating the pooling layers and dealing with the overfitting issues by initializing the weights into layers using ‘He Normal’ initialization along with the batch norm and global average pooling (GAP). Initially, we sharpen the input images using a Nimble filter, which results in maintaining the edges and fine details. After that, we employed the suggested BrainCDNet for the extraction of relevant features and classification. In this work, two different forms of magnetic resonance imaging (MRI) databases such as binary (healthy vs. pathological) and multiclass (glioma vs. meningioma vs. pituitary) are utilized to perform all these experiments.

**Results and discussion:**

Empirical evidence suggests that the presented model attained a significant accuracy on both datasets compared to the state-of-the-art approaches, with 99.45% (binary) and 96.78% (multiclass), respectively. Hence, the proposed model can be used as a decision-supportive tool for radiologists during the diagnosis of brain cancer patients.

## 1 Introduction

Brain tumors are characterized by the uncontrolled growth of cells within or near the brain. According to the American Cancer Society and the National Brain Tumor Foundation (NBTF) report, so far, more than 150 distinct brain tumors have been documented. Among them, glioma and meningioma tumors occur frequently, while pituitary tumors rarely happen. Typically, brain tumors are categorized into two groups: primary and secondary or metastatic. Primary brain tumors originate from the brain or its surroundings and are divided into benign (non-cancerous) or malignant (cancerous) (Rasheed et al., 2023). Tumors that do not have active cells and have less effect on human life are called benign. Meningioma and most pituitary tumors are benign. Tumors that contain active cells and highly impact human life are called malignant. Gliomas (astrocytoma, ependymoma, and oligodendroglioma) are malignant tumors. To identify these tumors, we require an adequate radiological examination and estimation. Physicians utilize medical imaging or scanning techniques, such as X-ray, computed tomography (CT), and magnetic resonance imaging (MRI) to meet this criterion. Among them, MRI is a frequently used non-invasive scanning procedure for identifying the abnormalities of brain tissues since it produces high spatial resolution images and is safe from radiation. Hence, it is most suitable for all subjects, such as children, adults, pregnant women, etc. In addition, MRI yields accurate visualization of anatomical structures in the human body, especially the soft tissues of the brain. Based on these details, radiologists or doctors will quickly decide to provide appropriate treatment to the patient, such as radiotherapy, chemotherapy, and surgery.

According to the Brain Cancer Statistics 2019 (Ilic and Ilic, 2023), 347,992 new cases (187,491 males and 160,501 females) are registered across the globe. Among them, 246,253 (138,605 males, 107,648 females) died from brain cancer. These statistics show that, in males, the incidence and mortality are higher than in females. The incidence rate for males is 4.8/100,000, and for females is 3.6/100,000, while the mortality rate in males is 3.9/100,000, and in females is 2.6/100,000. Worldwide, European countries have higher incidence and mortality rates (incidence rate: 7.9/100,000 in males and 5.5/100,000 in females; mortality rate: 5.4/100,000 in males and 3.5/100,000 in females).

From the statistical analysis, we conclude that early diagnosis of brain tumors is crucial in improving a patient’s lifespan. However, manually inspecting these MRI images for longer periods of time is tedious and prone to errors. Therefore, computer-aided diagnosis plays a vital role in assisting clinicians. Hence, in this work, we proposed a novel approach called BrainCDNet.

### 1.1 Highlights of this study

In this study, first, we exploited a Nimble filtering algorithm to highlight edge details within the image. Following this, we developed a novel deep neural network called BrainCDNet to extract meaningful features by addressing issues encountered in existing approaches, such as training parameters and network stability. The internal architecture of the suggested network is designed to enhance model accuracy by adjusting hyperparameters such as optimizer, learning rate, epochs, batch size, and the number of layers. Our model is implemented on binary (healthy vs. pathological) and multiclass (glioma vs. meningioma vs. pituitary) classification problems using 5-fold cross-validation and hold-out. The proposed framework achieved optimal performance with an accuracy of 99.45% (binary) and 96.78% (multiclass). Experimental outcomes demonstrate that the presented technique achieves better classification accuracy with fewer learning parameters (997,123, including 994,563 trainable and 2,560 non-trainable) than existing approaches.

## 2 Related works

For a past two decades, researchers and scientists focus on the classification of brain tumors from MRI images by adopting machine learning (ML), and deep learning (DL) mechanisms. In this section, we outline a few recently developed approaches.

Islam et al. (2021) proposed an enhanced brain tumor detection approach using super pixels and principal component analysis (PCA)-based feature extraction followed by template-based k-means (TKM) clustering. Through this sequence of steps, they attained 95% accuracy. Demir et al. (2023) suggested a deep learning (DL)-based model with an accuracy of 99% using MobileNetV2, ReliefF feature selection, and k-nearest neighbors (KNN). With the help of pre-trained convolutional neural networks (CNN) such as VGG-16, Srinivas et al. (2022) implemented a deep transfer learning framework that obtained 86.04% classification accuracy.

Shanthi et al. (2022) developed an optimized hybrid CNN (OHCNN) methodology using CNN followed by long short-term memory (LSTM) and adaptive RIDER optimization (ARO). The CNN-LSTM-ARO-based model generated an accuracy of 97.25%. Reddy et al. (2023) outlined a diagnosis model that utilized local texture features, blue monkey extended bald edge optimization (BMEBEO), and deep belief network (DBN) followed by Bi-LSTM (DBN-LSTM). The presented technique obtained an accuracy of 92.61%.

Vankdothu and Hameed (2022) offered an automated brain tumor detection approach with an accuracy of 95.17% for identifying the pathological behavior of MRI images. The proposed framework includes segmentation by improved k-means clustering (IKMC), feature extraction using gray-level co-occurrence matrix (GLCM), and classification based on recurrent CNN (RCNN). Zhu et al. (2023) developed a novel architecture: ResNet-based bat extreme learning machine (RBELM) to discriminate between normal and abnormal brain MRI images, gaining 99% detection accuracy.

Mijwil et al. (2023) suggested a MobileNet V1-based DL model to detect brain tumors from MRI images, and they yielded 97.3% accuracy. Rahman and Islam (2023) developed a parallel deep CNN (PDCNN) architecture to diagnose brain MRI tumors, and they attained 97.33% accuracy. To classify brain tumors into malignant and benign, Mehrotra et al. (2020) proposed an artificial intelligence (AI) based DL methodology with an accuracy of 99.04%.

Nanda et al. (2023) presented a new hybrid model that utilized a saliency KMC, social spider optimization, and radial basis neural network (RBNN). Through this process, the authors yield 92% accuracy. Kibriya et al. (2023) implemented an ensemble model by concatenating VGG-16 and GLCM features. Later, these features were fed to a support vector machine (SVM) to detect the pathological behavior of brain MRI images. The presented ensemble architecture attained 99.03% accuracy.

Abiwinanda et al. (2019) attempted to classify MRI-based brain tumors by developing an optimized CNN architecture. By this network, the authors achieved a detection rate of 84.19%. Pashaei et al. (2018) extracted relevant features using CNN and then employed a kernel-based extreme learning machine (KELM) for detecting brain tumors. Here, the suggested approach attained 93.68% accuracy.

Anaraki et al. (2019) proposed a CNN and genetic algorithm (GA) based model to grade the brain MRI images into glioma, meningioma, and pituitary. The experimental results of the presented model reveal that they attained 94.2% accuracy. Afshar et al. (2019) offered a modified CapsNet to analyze brain tumors from MRI images. Through this model, the authors achieved better performance with 90.89% accuracy in comparing conventional CNN approaches.

To implement an accurate brain tumor identification model, Gumaei et al. (2019) utilized a hybrid feature extraction framework such as PCA followed by gradient image descriptor (PCA-GIST) and regularized ELM (RELM). Here, the authors gained maximum accuracy with a 94.23% value. Swati et al. (2019) used a pre-trained CNN, namely VGG-19, and block-wise fine-tuning to distinguish MRI-based brain tumors. Based on this architecture, they achieved 94.82% classification accuracy.

Das et al. (2019) described a customized CNN-based strategy to interpret brain MRI images that achieved a significant accuracy of 94.39%. Kurmi and Chaurasia (2020) outlined an automated prognosis system with 92.6% accuracy based on hand-crafted features, neighborhood component analysis (NCA), and multilayer perceptron (MLP). Noreen et al. (2021) designed a hybrid technique through Xception and ensemble techniques. To improve the performance of the model, the authors further employed fine-tuning. Using this idea, they yield 94.34%.

Mukherkjee et al. (2022) offered a DL-based framework using aggregation of generative adversarial networks (AggrGAN) and ResNet-152 to identify the type of brain tumor. By making use of this technique, they obtained 93.88%. Deepak and Ameer (2023) utilized a deep feature fusion and majority voting mechanism to categorize brain tumors from the imbalanced MRI database. Here, the suggested approach attained 95.4% accuracy. To improve the performance of brain tumor diagnosis methodology, Khan et al. (2023) developed a hybrid network with 95.10% accuracy using DenseNet 169 and ML frameworks such as random forest (RF), SVM, and XGBoost.

## 3 Materials and methods

**Figure 1:**
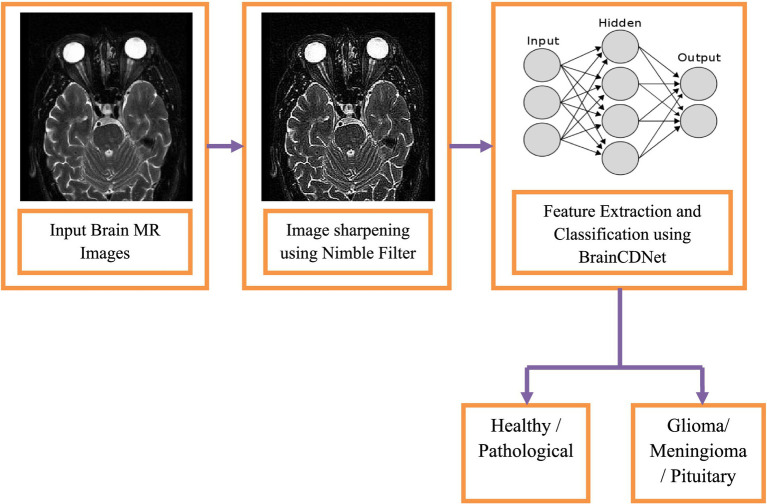
Block diagram of the proposed model.

This section describes the dataset used in the work and the relevant steps involved in the proposed model to identify the pathology of brain tumors using MRI images. Figure 1 indicates the flow diagram of the suggested BrainCDNet. Subsequent sections describe each block of Figure 1.

### 3.1 Dataset

**Figure 2:**
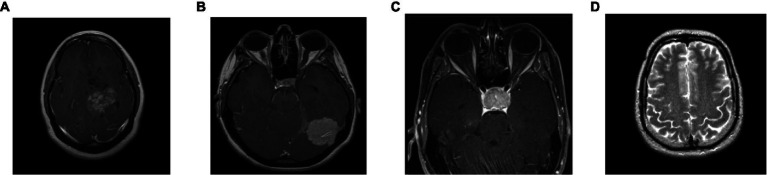
Sample brain MRI images: **(A)** glioma; **(B)** meningioma; **(C)** pituitary; **(D)** healthy.

To analyze the presented framework, we consider the two brain MRI image dataset scenarios: binary (healthy vs. pathological) and multiclass (glioma vs. meningioma vs. pituitary). The first scenario comprised 2,376 T2-weighted MRI images, including 1746 pathological (glioma, Sarcoma, meningioma, and Alzheimer’s) and 630 healthy images (Kaggle, n.d.). The second scenario constituted 2,764 T1-weighted contrast-enhanced MRI images with 926 glioma, 937 meningioma, and 901 pituitary gland tumor images (MRI, n.d.). Figure 2 shows the sample images used in this work. All of these images have varied resolution sizes; however, to feed these images to the proposed deep net, we resize them to 224 × 224 × 3.

### 3.2 Image sharpening

Image sharpening is a digital image processing technique used to enhance the contrast and detail of an image. The goal of sharpening is to improve the visual perception of the image by enhancing the edges, contours, texture, and some fine details that may have been blurred or softened during image capturing or processing. Over the last few decades, various approaches have been developed to fulfill the requirements of image sharpening. Unsharp masking, Laplacian sharpening, deconvolutional sharpening, and frequency-domain filtering techniques are more popular among them. However, image sharpening is an open problem for researchers due to the over-sharpening (Toh and Isa, 2011) and amplification of image noise (Sheppard et al., 2004). To minimize these problems, we used a Nimble filter (Zohair, 2018), developed recently and the processes is as follows:

1. Initially, generate a blurred version of the original image, *I(m,n)*, by estimating the average of the horizontal and vertical shifts for a source image, then multiply by a scaling factor α. The resultant image includes the spatial details of original image.
2. Secondly, multiply the original image with a scaling factor α.
3. Finally, add outcomes of steps 1 and 2 to obtain the resultant sharpened image.

**Figure 3:**
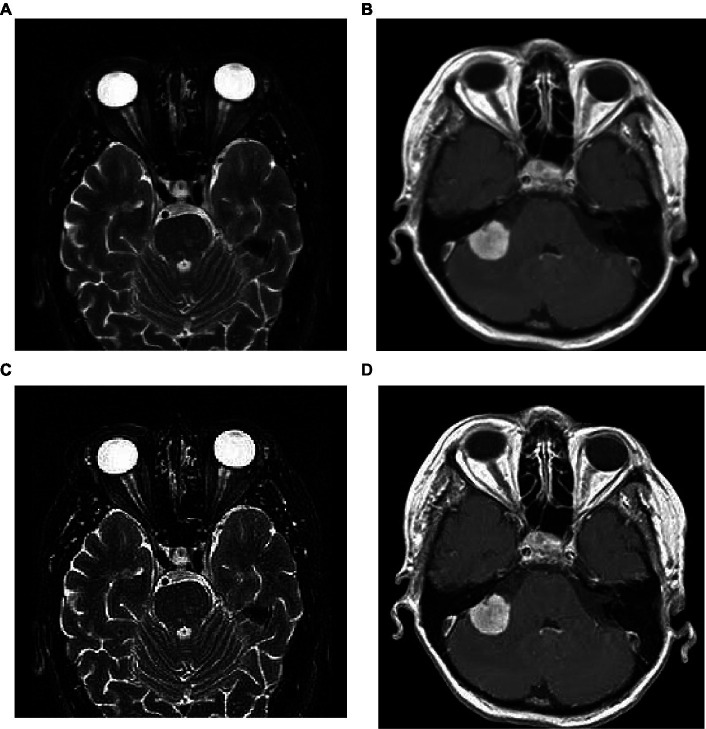
Image sharpening using Nimble filtering: **(A,B)** original images; **(C,D)** sharpened images.

The mathematical characterization of the suggested Nimble filter is described in the Equation 1 as follows:


(1) $$\begin{array}{l} S\left(m,n\right)=\alpha \times I\left(m,n\right)\\ \;+\left[\left(1-\alpha \right)\times \left(\frac{I_{m,n+1}+I_{m,n-1}+I_{m+1,n}+I_{m-1,n}}{4}\right)\right] \end{array}$$

where, *S(m,n)* is the sharpened image; *m,n* are the spatial coordinates; α is a scaling factor that controls the amount of sharpness enhancement, and which is always greater than 1. In this work, we consider α as 2 and the corresponding implications of image sharpening are represented in Figure 3. From this figure, we observed that due to the image sharpening, we highlighted the patterns (see Figure 3C) and removed the blurring (see Figure 3D) from the original images.

### 3.3 The BrainCDNet architecture

**Figure 4:**
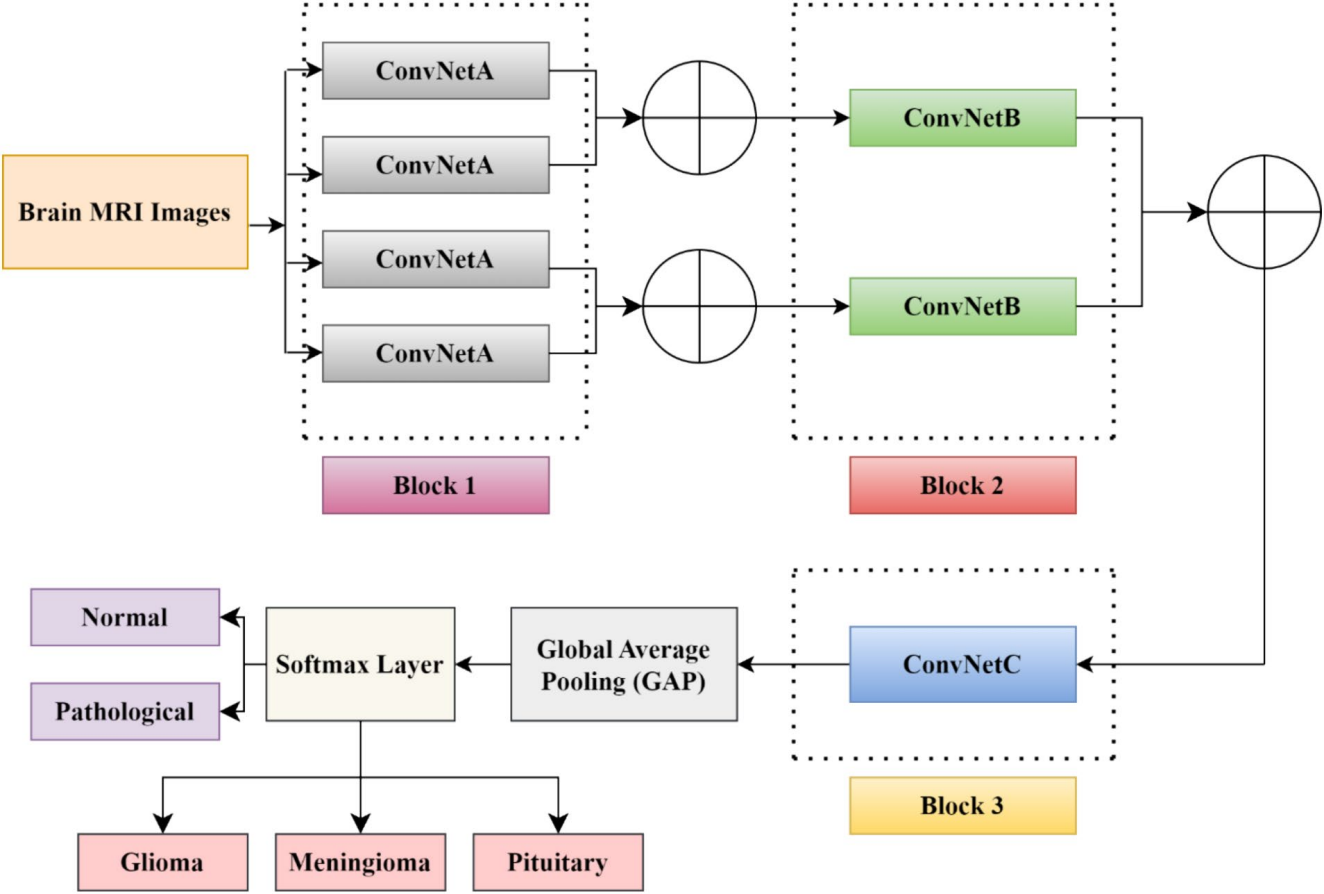
The suggested BrainCDNet architecture.

In this study, we developed a BrainCDNet model by concatenating the pooling layers, which is shown in Figure 4. The suggested architecture mainly includes three CNN blocks (blocks 1 to 3), global average pooling, and a softmax layer.

The first block (or block 1) consists of four ConvNetA architectures, which can be used as a feature extractor. Each ConvNetA includes a 3 × 3 convolutional layer with 64 filters and a stride 2, scaled exponential linear unit (SELU), batch normalization, and 2 × 2 max-pooling with stride 2. Features obtained from each ConvNetA are concatenated using Equation 2 as follows (Barzekar and Zeyun, 2022):


(2) $$F\left(x,y\right)=x_{p,q,c_{m}}⊕y_{p,q,c_{n}}=C_{p,q,c_{m}+c_{n}}$$

where, *(p,q)* are the dimensions of ConvNet; *x* and *y* denotes the feature maps; *(c_m_,c_n_)* represents the number of channels on each ConvNet output, ⨁ illustrates the concatenation operator.

The above process is applied to the top two and the bottom two ConvNetA architectures. The corresponding feature maps obtained from the first block are fed to the second block CNN framework.

The second block (or block 2) incorporates two ConvNetB architectures. Each ConvNetB includes 2 convolutional layers with a kernel size of 3 × 3 and 1 × 1, stride 1, and 128 filters. Here, by the 1 × 1 convolution, we relatively minimize the model’s computational complexity and reduce the significant number of feature maps. Later on, after 3 × 3 convolutions, we placed a SELU activation and batch normalization. Similarly, after 1 × 1 convolutions, we introduced a SELU activation and batch normalization followed by 2 × 2 max-pooling with stride 2. The outcomes of both ConvNet B architectures are concatenated by Equation (2) , and the resultant feature maps feed as input to block 3.

The third block (or block 3) contains one CNN architecture namely, ConvNetC, which has the following configurations: One convolutional layer with a filter size of 3 × 3, stride 1, and 256 filters, followed by Gaussian error linear unit (GELU) and batch normalization. In addition, we incorporated a 1 × 1 convolution with the same configurations. Afterward, the features obtained from the third block are summarized by a global average pooling (GAP). By this pooling layer, we can reduce the number of training parameters and also prevent overfitting issues. Finally, these summarized features are fed to the softmax layer to classify the brain MRI images into two categories: healthy vs. pathological and glioma vs. meningioma vs. pituitary. The hyperparameters of the BrainCDNet architecture is presented in Table 1.

**Table 1:** The hyperparameter values used for modeling the BrainCDNet architecture.

Optimizer	Adam
Hyper-parameter	Values
Learning rate	0.001
Batch size	64
Epochs	50

## 4 Results and discussions

This section presents the simulation results of the proposed method, as shown in Figure 1. As mentioned in the previous section, the MRI images are first preprocessed using the Nimble filter for better visualization and diagnosis. Later, we followed two classification scenarios. In the first scenario, we considered 2376 T2-weighted MRI images from the healthy and pathological subjects. The experimentation on this group discriminates only between these two classes. In scenario 2, we considered 2764 T1-weighted MRI images from three classes, namely glioma, meningioma, and pituitary gland tumor images. This set of experiments classifies these three pathological MRI images.

### 4.1 Performance metrics

The choice of performance metrics is crucial for any ML or DL-based models for quantified analysis. In this work, we adopted the most often used metrics in the literature (Srinivas et al., 2022): true positive rate (TPR)/Sensitivity, true negative rate (TNR)/Specificity, positive predictive value (PPV)/Precision, F-score, area under curve (AUC), and accuracy. For better performance, all of these values must be high.

#### 4.1.1 Scenario 1 (binary classification)

Before executing any DL model, the first step is to separate the dataset into training and test sets for proper validation. Therefore, the selection of a validation scheme is a significant step. In this work, we performed two types of validation schemes, namely, K-fold cross-validation and a holdout, to estimate the performance of the proposed method. The whole data will be divided into approximately K-portions in the K-fold scheme. Later, one portion of the data will be kept for testing, and the remaining K-1 portion will be used for training the model. This process repeats for K times, and a different test set will be used for each run. Finally, the average result of all these K runs is considered for the model. Besides, the holdout scheme will randomly divide the dataset into training and testing datasets by ensuring data from all classes is available in both datasets. Here, we considered K = 5 in the prior validation scheme and two holdout cases (Case 1: 75% of data for training and 25% for testing. Case 2: 85% of data for training and 15% for testing).

**Figure 5:**
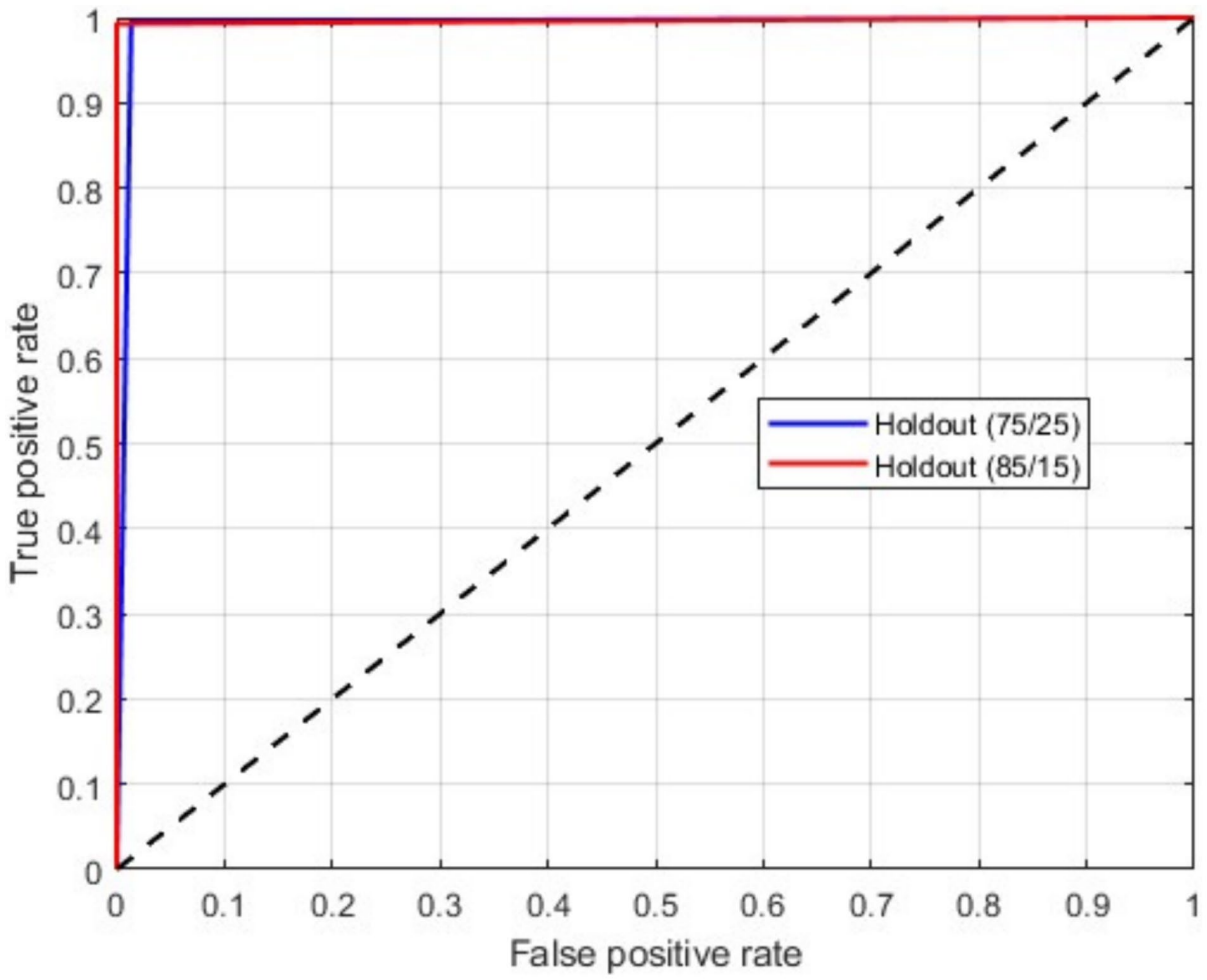
ROC curve for a binary classification using holdout method.

Table 2 presents the binary classification results with the proposed BrainCDNet using 5-fold cross-validation and two holdout schemes mentioned above. Our method gives better results in all validation scenarios, and the most significant results are highlighted in bold font. Figure 5 represents the receiver operating characteristics (ROC) of the holdout method. From this we observed that holdout (85/15) method has a wider ROC curve compared to the holdout (75/25), which indicates better classification performance.

**Table 2:** Evaluation measures of the presented binary classification model.

Validation	Scenario	Evaluation metrics (%)					
		TPR	TNR	PPV	F-Score	AUC	Accuracy
K-FCV	5-FCV	99.61	99.06	99.65	99.62	99.33	99.44
Holdout	75/25	99.55	98.67	99.55	99.55	99.11	99.33
	85/15	99.27	100	100	99.63	99.63	99.45

#### 4.1.2 Scenario 2 (multiclass classification)

**Figure 6:**
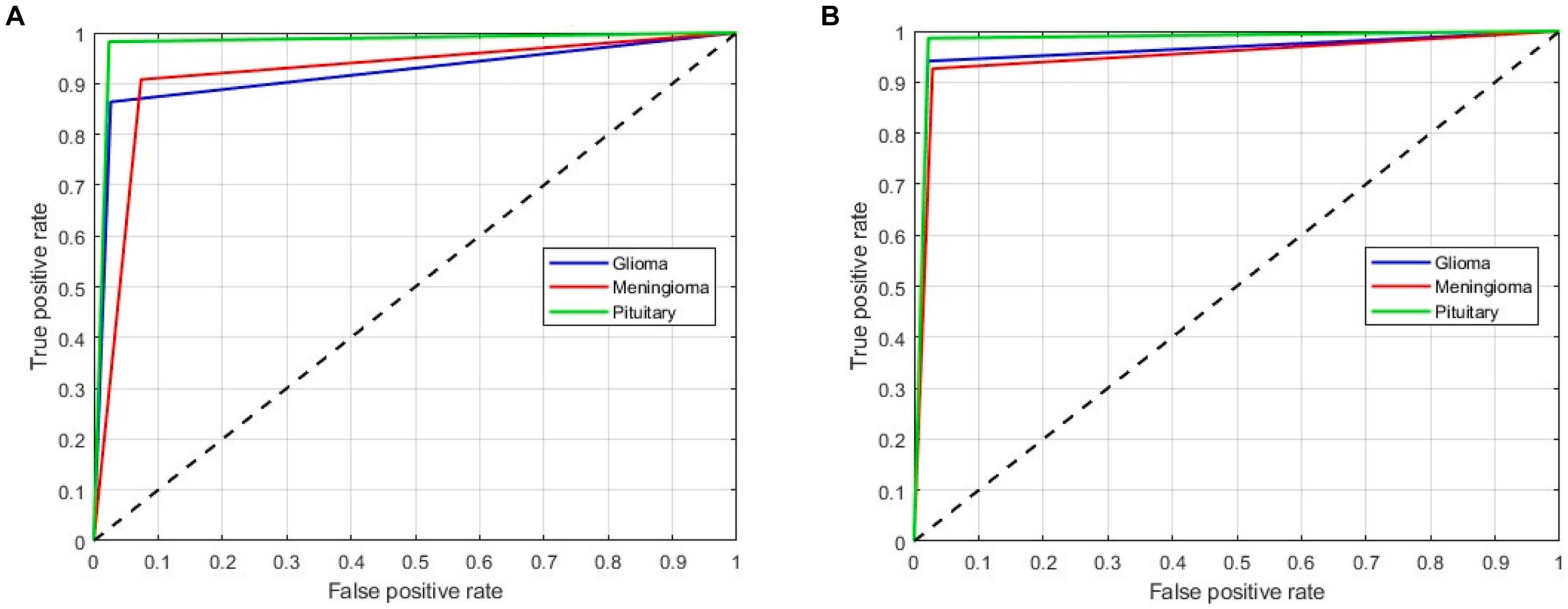
ROC curves for a multiclass classification using holdout method: **(A)** Holdout (75/25); **(B)** Holdout (85/15).

As mentioned above, in this scenario, we considered a multiclass classification. The given MRI images are distinguished between the three pathological classes, glioma, meningioma, and pituitary. It is one of the essential models for preparing for real-time situations. Table 3 presents the simulation results of this scenario. The table shows that the proposed approach yields a better result by more than 90% in all validation schemes. However, the holdout scheme is dominating among them. The average results of each scheme are presented in boldface. Another important observation from the table is that our approach provides higher metric values for each class. Figure 6 illustrates the ROC curves for the multiclass classification using holdout method. From these, we observed that compared to the other primary tumors, pituitary brain tumors are significantly classified.

**Table 3:** Evaluation measures of the suggested multiclass classification model.

Validation	Scenario	Class	Evaluation metrics (%)					
			TPR	TNR	PPV	F-Score	AUC	Accuracy
K-FCV	5-FCV	Glioma	93.20	93.79	88.03	90.48	93.28	93.48
		Meningioma	99.32	97.89	95.93	97.56	98.61	98.37
		Pituitary	88.12	98.58	96.91	92.26	93.35	95.04
		Average	93.55	96.75	93.62	93.43	95.08	95.63
Holdout	75/25	Glioma	86.38	97.37	94.42	90.26	91.87	93.63
		Meningioma	90.78	92.65	85.89	88.27	91.71	92.04
		Pituitary	98.24	97.62	95.32	96.76	97.93	97.83
		Average	91.8	95.88	91.88	91.76	93.84	94.5
	85/15	Glioma	94.12	97.85	95.52	94.81	95.98	96.62
		Meningioma	92.64	97.13	94.03	93.33	94.88	95.66
		Pituitary	98.60	97.8	95.92	97.24	98.2	98.07
		Average	95.12	97.6	95.16	95.13	96.35	96.78

### 4.2 Ablation study

To evaluate the contribution of the image enhancement using Nimble filter to the overall performance of our model, we conducted an ablation study by training and evaluating our model with and without using the Nimble filter. The Tables 2, 3 presents the results with Nimble filter, and the Tables 4, 5 presents the results without using Nimble filer.

**Table 4:** Evaluation measures of the presented binary classification model (without Nimble filter).

Validation	Scenario	Evaluation metrics (%)					
		TPR	TNR	PPV	F-Score	AUC	Accuracy
K-FCV	5-FCV	99.14	97.62	99.08	99.10	98.38	98.69
Holdout	75/25	96.52	99.38	99.76	98.11	97.95	97.31
	85/15	96.5	100	100	98.22	98.25	97.48

**Table 5:** Evaluation measures of the proposed multiclass classification model (without Nimble filter).

Validation	Scenario	Class	Evaluation metrics (%)					
			TPR	TNR	PPV	F-Score	AUC	Accuracy
K-FCV	5-FCV	Glioma	87.14	95.52	91.43	86.78	91.33	92.73
		Meningioma	80.4	94.84	89.51	83.73	87.61	89.94
		Pituitary	98.94	92.83	87.85	92.99	95.88	94.9
		Average	88.54	95.52	88.67	88.80	91.61	90.48
Holdout	75/25	Glioma	89.91	94.38	88.74	86.59	92.15	92.91
		Meningioma	56.84	98.67	95.80	71.35	77.75	84.08
		Pituitary	100	79.74	70.03	82.37	89.87	86.25
		Average	82.25	90.93	84.86	80.10	86.59	87.74
	85/15	Glioma	75.75	100	100	86.20	87.87	92.28
		Meningioma	98.69	84.73	79.05	87.79	91.71	89.87
		Pituitary	93.84	99.29	98.38	96.06	96.57	97.59
		Average	89.43	94.67	92.48	90.02	92.05	93.25

Comparing the metric values in Tables 2, 4, and similarly, Tables 3, 5, it is evident that the results are significantly improved when using the Nimble filter. Specifically, the accuracy of the model is higher when the Nimble filter is applied. These results indicate that the Nimble filter plays a crucial role in improving the performance of our model.

### 4.3 Discussion

To understand the efficacy of our proposed method, we conducted a comparative analysis with the existing state-of-the-art. We compared our approach with existing methods by scenario (binary and multiclass) for fair analysis. Table 6 shows the results of the existing literature and proposed approach for binary classification, and Table 7 is about multiclass classification. In these tables, we compared the metric accuracy for all the methods as it is a standard measure used by all the mentioned works.

**Table 6:** Comparison between the proposed and existing binary classification models.

Method	Evaluation measures (%)		
	TPR	TNR	Accuracy
TKM-PCA (Islam et al., 2021)	97.36	100	95
MobileNetV2-KNN (Demir et al., 2023)	100	97.96	99
VGG-16 (Srinivas et al., 2022)	83.33	89.47	86.04
CNN-LSTM (Shanthi et al., 2022)	96.81	97.64	97.25
DBN-LSTM (Reddy et al., 2023)	95.64	85.83	92.61
GCM-RCNN (Vankdothu and Hameed, 2022)	98.42	89.28	95.17
RBELM (Zhu et al., 2023)	100	95	99
MobileNetV1 (Mijwil et al., 2023)	97	96.1	97.3
PGCNN (Rahman and Islam, 2023)	95.65	100	97.33
AlexNet (Mehrotra et al., 2020)	99.30	98.50	99.04
RBNN (Nanda et al., 2023)	93	91	92
VGG-GLCM-SVM (Kibriya et al., 2023)	98.67	99.37	99.03
BrainCDNet (The Proposed)	99.27	100	99.45

**Table 7:** Comparison between the proposed and existing multiclass classification models.

Method	Evaluation measures (%)		
	TPR	TNR	Accuracy
Optimized CNN (Abiwinanda et al., 2019)	-	-	84.19
CNN-KELM (Pashaei et al., 2018)	91.43	-	93.68
CNN-GA (Anaraki et al., 2019)	94.2	97.1	94.2
CapsNet (Afshar et al., 2019)	-	-	90.89
PCA-GIST-RELM (Gumaei et al., 2019)	93.46	97.05	94.23
VGG-19 (Swati et al., 2019)	94.25	94.69	94.82
Customized CNN (Das et al., 2019)	92.64	97.02	94.39
NCA-MLP (Kurmi and Chaurasia, 2020)	-	-	92.6
Inception-v3-Ensemble (Noreen et al., 2021)	92.33	**-**	94.34
AggrGAN-ResNet 152 (Mukherkjee et al., 2022)	-	-	93.88
CNN- Majority Voting (Deepak and Ameer, 2023)	94.93	-	95.4
DenseNet-169 (Khan et al., 2023)	95	94	95.10
BrainCDNet (The Proposed)	95.12	97.6	96.78

The hyphen (−) mark denotes the results are not reported in that article.

Table 7 shows the results of the multiclass classification. It is evident from the results that the proposed work achieved considerable improvement over the other works. The discussion on the proposed work with the compared works is given below.

Tables 6, 7 demonstrates that most of the existing studies achieved over 95% accuracy in MRI image classification. However, these studies suffer from several limitations.

The study in Islam et al. (2021) utilized a combination of datasets, including one mentioned in (Kaggle, n.d.), yet their dataset for classification purposes consisted of only about 40 MRI images, which is nearly 60 times smaller than the dataset used in our research. Similarly, studies in Shanthi et al. (2022), Srinivas et al. (2022), Demir et al. (2023), Reddy et al. (2023), Zhu et al. (2023), Mehrotra et al. (2020), and Nanda et al. (2023) employed deep neural networks but were constrained by relatively small datasets, ranging from 100 to 1,000 MRI images. To fully exploit the effectiveness of deep networks, it is essential to train them with extensive data. While the work mentioned in Vankdothu and Hameed (2022) used a substantial dataset of 2,870 images, the authors first extracted handmade features before applying them to the RNN. In contrast, our proposed algorithm bypasses this manual feature extraction process, achieving the desired result more efficiently. In another study (Mijwil et al., 2023), the authors grouped MRI images of glioma, pituitary, and meningioma tumors into one category, totaling 7,788 images, and treated 2,500 images as no tumor images. Despite employing a considerable number of MRI images, they followed only a binary classification scheme. In contrast, our proposed approach considered two distinct scenarios for improved predictive analysis. The study (Rahman and Islam, 2023), utilized three datasets for classification, employing a significant number of MRI images and various holdout schemes for validation. Despite achieving a high accuracy rate of 97.33%, which is less than 2% lower than our approach, the employed deep neural network architecture required more parameters to implement. Our proposed BrainCDNet architecture reduces the number of training parameters and enhances network stability. Finally, in another study (Kibriya et al., 2023), the authors achieved a 99% classification result by combining the features of a deep network and GLCM and using SVM for classification. However, this approach does not constitute a fully adaptive feature extraction scheme like our proposed BrainCDNet.

### 4.4 Overall remarks

In contrast to many existing works in the literature that primarily focus on binary classification or multiclassification schemes, our proposed approach demonstrates improved results for both of these cases. While many existing studies relied on single cross-validation analysis, we provided results for three different validation schemes, enhancing the robustness and reliability of our findings. Compared to many existing studies, we considered a larger MRI image dataset, allowing for more comprehensive analysis and evaluation of our proposed approach. Our proposed BrainCDNet scheme is implemented in a block-wise manner, significantly reducing the number of trainable parameters. This reduction in parameters not only streamlines the implementation process but also enhances the efficiency and stability of the network.

Besides, the results of the ablation study demonstrate the importance of the image enhancement filter in improving the performance of our model. By enhancing the input images before feeding them into the model, we were able to achieve higher accuracy and better image quality. This confirms the effectiveness of our proposed approach for image enhancement using machine learning. Medical images often rely on fine details and clear edges for accurate diagnosis. The Nimble Filter is designed to enhance these aspects effectively. Traditional sharpening filters can amplify existing noise in images. The Nimble Filter aims to achieve sharpening while minimizing the impact on noise levels.

## 5 Conclusion

In conclusion, developing accurate and efficient methods for detecting and classifying brain tumors is paramount in improving patient outcomes. This study introduces BrainCDNet, a novel DL architecture for brain tumor classification using MRI. By leveraging advanced techniques such as Nimble filtering for image sharpening, batch normalization for addressing overfitting, and GAP for feature extraction, BrainCDNet demonstrates a good performance on both binary (healthy vs. pathological) and multiclass (glioma vs. meningioma vs. pituitary) MRI databases.

The results presented in this study showcase the effectiveness of BrainCDNet in accurately identifying and classifying brain tumors, achieving an impressive accuracy of 99.45% for binary classification and 96.78% for multiclass classification. These findings highlight the potential of DL-based approaches in medical image analysis and underscore the importance of early detection and precise diagnosis of brain cancer where timely intervention is critical.

## Data availability statement

Publicly available datasets were analyzed in this study. This data can be found at: https://www.kaggle.com/datasets/navoneel/brain-mri-images-for-brain-tumor-detection; https://www.kaggle.com/datasets/sartajbhuvaji/brain-tumor-classification-mri.

## Ethics statement

Ethical approval was not required for the studies involving humans because publicly available datasets were used.

## Author contributions

KRe: Writing – original draft. KRa: Writing – original draft. RD: Writing – review & editing. VK: Writing – review & editing.
